# Mechanically Strong and Electrically Conductive Polyethylene Oxide/Few-Layer Graphene/Cellulose Nanofibrils Nanocomposite Films

**DOI:** 10.3390/nano12234152

**Published:** 2022-11-23

**Authors:** Mei Li, Meijie Xiao, Qunhao Wang, Jian Zhang, Xiaolin Xue, Jiangqi Zhao, Wei Zhang, Canhui Lu

**Affiliations:** 1 State Key Laboratory of Polymer Materials Engineering, Polymer Research Institute at Sichuan University, Chengdu 610065, China; 2 Advanced Polymer Materials Research Center of Sichuan University, Shishi 362700, China

**Keywords:** cellulose nanofibrils, polyoxyethylene, graphene, nanocomposite films, electrical conductivity

## Abstract

In this work, a cellulose nanofibrils (CNFs)/few-layer graphene (FLG) hybrid is mechanically stripped from bamboo pulp and expanded graphene (EG) using a grinder. This strategy is scalable and environmentally friendly for high-efficiency exfoliation and dispersion of graphene in an aqueous medium. The in situ-generated CNFs play a key role in this process, acting as a “green” dispersant. Next, the obtained CNFs-FLG is used as a functional filler in a polyoxyethylene (PEO) matrix. When the composition of CNFs-FLG is 50 wt.%, the resultant PEO/CNFs-FLG nanocomposite film exhibits a Young’s modulus of 1.8 GPa and a tensile strength of 25.7 MPa, showing 480% and 260% enhancement as compared to those of the pure PEO film, respectively. Remarkably, the incorporation of CNFs-FLG also provides the nanocomposite films with a stunning electrical conductivity (72.6 S/m). These attractive features make PEO/CNFs-FLG nanocomposite films a promising candidate for future electronic devices.

## 1. Introduction

Carbon nanofillers, such as graphene, carbon nanotubes and carbon black [1,2,3], have been widely applied in polymer nanocomposites due to their special structure, thermal stability, electrochemical stability and excellent mechanical properties. In particular, the 2D nanocarbon of graphene has shown great promise in the preparation of high-performance polymer nanocomposites thanks to its extraordinary properties, such as a high electrical conductivity of ~106 S cm^−1^ and a high modulus of ~100 GPa [4,5]. Meanwhile, improvement in the physicochemical properties of graphene nanocomposites depends on the distribution of graphene in the polymer matrix as well as interfacial bonding between the filler and the polymer matrix. However, conventional graphene-based nanocomposites usually suffer from the poor dispersion of graphene in the matrix owing to the strong tendency of graphene to form agglomerates [6]. To tackle this problem, various methods have been developed, including surface modification [7], incorporation of organic salts to assist dispersion [8] and so on. Currently, there are several approaches to preparing graphene, including chemical vapor deposition [9], arc discharge [10] and chemical conversion [11]. Among them, chemical oxidation/reduction of different carbon sources (e.g., graphite oxide [12], expandable graphite (EG) [13] and sieved graphite powder [14]) is one of the most commonly practiced methods. However, this method exhibits some obvious drawbacks which greatly limit application on a large scale. For example, the chemical reaction destroys the electron clouds of graphene, which reduces its inherent conductivity. Moreover, the use of concentrated acid and toxic chemicals increases the handling risk and health hazard.

Liquid phase exfoliation (LPE) [15] is a physical method capable of peeling off graphene directly from graphite powder. Compared with the chemical methods, LPE has advantages of simplicity, high efficiency, low cost and environmental friendliness. More importantly, the graphene prepared by LPE usually has fewer defects in its structure, which is highly desired for many end-uses. In addition, because water is an economic and “green” solvent, great research efforts have been devoted to LPE of graphene using water as the liquid medium. However, the surface energy mismatch between water and graphene always results in aggregation and restacking of the graphene sheets. Consequently, various stabilizers, such as aromatic compounds [16], surfactants [17] and polymers [18], have been applied, aimed at addressing this issue.

Cellulose nanofibrils (CNFs) extracted from plant fibers are nanofibrous materials with diameters in the range of 1 to 100 nm and lengths of several microns [19]. They have been proved to be an outstanding reinforcing filler for polymer composites with many other unique properties, such as low density, small size, renewability, biodegradability, high specific surface area, good biocompatibility and mechanical properties [20,21]. Specifically, the presence of -OH and -CH on their molecular chains makes them both hydrophilic and hydrophobic [22]. This amphiphilic property of CNFs has been frequently utilized to stabilize various nanoparticles [23,24], preventing their agglomeration and restacking in an aqueous medium [25]. It is envisaged that during a wet co-milling process of cellulose and graphite, both CNFs and graphene can be simultaneously peeled off from their precursors. Meanwhile, the CNFs and graphene interact with each other through hydrogen bonding and hydrophobic–hydrophobic interaction [26], giving rise to a stable CNFs/graphene aqueous dispersion. In addition, the obtained hybrid nanofiller might synergistically reinforce polymer composites because of its excellent mechanical properties.

Polyoxyethylene (PEO) is a thermoplastic and water-soluble polymer with excellent biocompatibility, which is particularly attractive in coating [27], polyelectrolytes for energy harvesting and storage [28,29,30], etc. However, neat PEO materials intrinsically exhibit low mechanical performance and poor electrical conductivity. The incorporation of conductive filler to fabricate strong and conductive PEO composites will certainly broaden the application fields of PEO. For example, conductive PEO composites can be used for various sensing applications, such as humidity sensors [31], H_2_S gas sensors [32], toluene sensors [33,34] and acetone and ethanol sensors [35]. In addition, they also show promise in electrocatalysts, electromagnetic shielding, flexible electronics and so on [36,37,38,39].

Herein, we demonstrate a facile and scalable strategy to fabricate strong and conductive PEO nanocomposite films. First, hybrid CNFs/few-layer graphene (FLG) nanofillers with stable dispersion in water were prepared from bamboo pulp fibers and EG via wet co-milling in a grinder. Subsequently, the nanofillers were blended with a PEO solution to produce PEO/CNFs-FLG nanocomposite films. In this composite system, both the dispersion of graphene and the interfacial compatibility between filler and polymer matrix could be effectively improved. Furthermore, the effects of CNFs-FLG loading on the thermal, mechanical and electrical properties of the PEO nanocomposite films were comprehensively investigated.

## 2. Materials and Methods

### 2.1. Materials

Never-dried Moso bamboo pulp with a solid content of 25% was supplied by Yongfeng Paper Co., Ltd., Muchuan, China. Expandable graphene ((EG), KP251) was provided by ADT Carbonic Material Factory (Shijiazhuang, China) with a particle size of 50 mesh, a density of 2.05 g/cm^3^ and an onset exfoliation temperature of 150 °C. Polyethylene oxide ((PEO), viscosity-average molecular weight = 600,000) was purchased from Kelong Chemical Reagent Co., Ltd., Chengdu, China.

### 2.2. Preparation of CNFs-FLG Hybrid Nanofillers

The CNFs-FLG hybrid nanofillers were fabricated as follows. First, EG was expanded by microwave irradiation at 350 W for 20 s to obtain vermiform EG particles, following the literature [40]. Then, the water dispersion with a solid concentration of 1 wt.% and a weight ratio of bamboo pulp: EG = 5:5 was mechanically stirred for 12 h. After that, they were subjected to a superfine grinding machine (MKCA6-2, MasukoSangyo Co., Ltd., Saitama, Japan) at 1500 rpm to produce a CNFs-FLG dispersion, which was denoted as CNFs: FLG = 5:5. The clearance between the upper and lower disks was set at −10 μm, −50 μm and −100 μm. The suspension was treated for 10 cycles at each clearance sequentially.

### 2.3. Preparation of PEO/CNFs-FLG Composite Films

First, PEO was dissolved in deionized water at 80 °C to obtain a PEO solution with a concentration of 2 wt.%. Next, the solution was cooled to 60 °C and the CNFs-FLG dispersion (5:5 in weight) with a solid concentration of 1 wt.% was gradually added and mechanically stirred for 6 h. Finally, the PEO/CNFs-FLG mixture was poured into a polystyrene dish and placed in an oven at a temperature of 60 °C for 48 h to obtain the PEO/CNFs-FLG composite films. The compositions of the mixtures are provided in Table 1. The resultant PEO/CNFs-FLG composite films with different CNFs-FLG content were designated as PEO/CNFs-FLG 0%, PEO/CNFs-FLG 10%, PEO/CNFs-FLG 30%, PEO/CNFs-FLG 50% and PEO/CNFs-FLG 80% according to the addition of CNFs-FLG.

### 2.4. Characterization

The microscopic morphology of the CNFs-FLG sample was observed by optical microscope (UB200i; Oupu Optoelectronic Technology Co., Ltd., Chongqing, China). To prepare the sample, the CNFs-FLG aqueous dispersion was diluted and dropped on a glass slide, which was then covered with a coverslip to spread the dispersion evenly.

The morphologies of fractured samples were observed by scanning electron microscopy (FESEM, JEOL JSM-7500F, Tokyo, Japan) at 20 kV after sputtering a thin Au coating onto the sample surface. Fourier transform infrared spectroscopy (NICOLET 6700, Thermo Fisher Scientific, Waltham, MA, USA) was used to characterize the chemical composition of CNFs-FLG. In addition, high-resolution transmission electron microscopy (HRTEM, JEOL JEM-100CX, Tokyo, Japan) was used to analyze their micromorphology.

Mechanical properties were assessed by a universal material testing machine (INSTRON 5966, INSTRON, MA, USA) with a loading rate of 10 mm/min according to the standard GB/T1040-92. The samples were cut into strips with a width of 1 mm and length of 3 mm, while the thickness of the samples was measured by a thickness gauge. For each composite film, 5 parallel samples were tested, and the average value was presented. The resistance of the PEO/CNFs-FLG composite films were measured with a KDB-1 resistivity/box resistance tester (Guangzhou Kunde Technology Co., Ltd., Guangzhou, China) and the copper electrodes were linearly arranged. For each composite film, 10 points on the sample surface were randomly tested, and the average value was presented. The conductivity (σ) was calculated as follows:

ρ = Rs/t(1)

σ = 1/ρ(2)

where ρ is resistivity; Rs is square resistance; and t is the thickness of the film.

The melting behavior of the PEO/CNFs-FLG nanocomposite was recorded with a DSC204F1 differential scanning calorimeter (NETZSCH, Bavarian Asia, Germany). A sample of 5–10 mg was accurately weighed and placed into a crucible. The DSC curve was recorded from 30 °C to 150 °C at a rate of 10 °C/min. All samples were dried in a vacuum oven at 60 °C for 24 h before testing. The sample crystallinity (X) was calculated according to the following formula:

(3) $$X\;(\%)=\frac{\Delta H_{f}\;}{\Delta H_{f}^{0}}\;\times \;100\%$$

where Δ*H_f_* is the melting enthalpy of the system; Δ*H_f_*^0^ is the melting enthalpy for 100% PEO crystal—its value is 231.7 J/g; and W is the content of CNFs-ELG in the composite.

## 3. Results and Discussion

### 3.1. Stability of CNFs-FLG Aqueous Suspension

**Figure 1:**
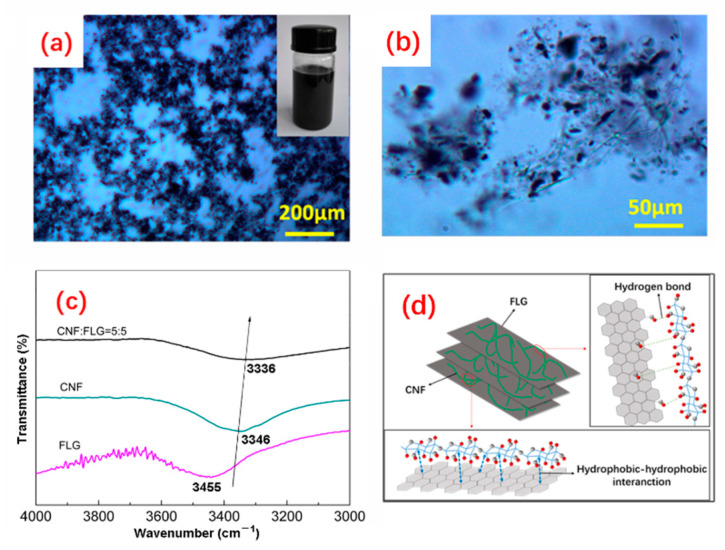
(**a**,**b**) Optical microscope images of CNFs-FLG. The inset is a digital photo of CNFs-FLG aqueous dispersion after 48 h resting; (**c**) FTIR spectra of FLG, CNFs and CNFs-FLG; (**d**) Schematic diagram of CNFs-FLG structure.

**Figure 2:**
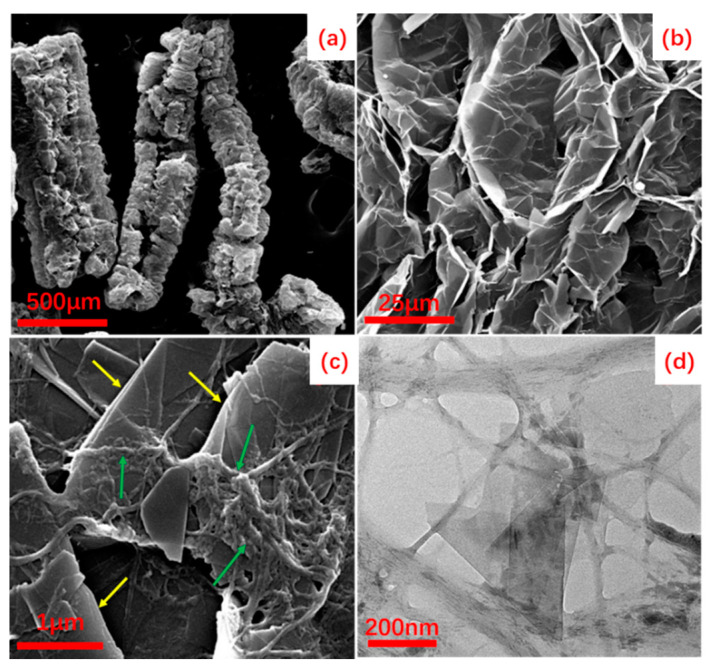
(**a**,**b**) SEM images of EG; (**c**) SEM image of CNFs-FLG; (**d**) TEM image of CNFs-FLG. The green and yellow arrows indicate CNFs and FLG, respectively.

The CNFs-FLG hybrid fillers were prepared by the wet co-milling of bamboo pulp and EG. As shown in the inset of Figure 1a, the CNFs-FLG aqueous dispersion demonstrated excellent stability even after 48 h of resting, and no noticeable aggregation was observed (Figure 1a,b). The FTIR spectra (Figure 1c) showed that the characteristic absorption for the -OH stretching vibration shifted to a lower wavenumber (from 3346 cm^−1^ to 3336 cm^−1^), suggesting that the -OH groups on CNFs could form hydrogen bonding with the oxygen-containing groups at the edge of defective FLG [41]. The synergistic effects of hydrogen bonding and hydrophobic–hydrophobic interaction between the CNFs and FLG made them highly dispersed in the aqueous medium, effectively hindering the agglomeration and restacking of the FLG [20,41]. The SEM image in Figure 2a shows that the EG had a porous worm-like structure. A magnified SEM image for the “worm” is provided in Figure 2b, in which the graphene layers can be distinguished. For the CNFs-FLG sample, the CNFs wrapped and adhered well to the FLG surface (Figure 2c). Moreover, the transparent graphite sheets could be clearly observed in TEM images (Figure 2d), indicating that the EG had been exfoliated to thin sheets during the wet co-milling [41]. Accordingly, a schematic diagram is provided in Figure 1d, showing the CNFs-FLG structure and their interactions.

### 3.2. Morphology of PEO/CNFs-FLG Nanocomposite Films

**Figure 3:**
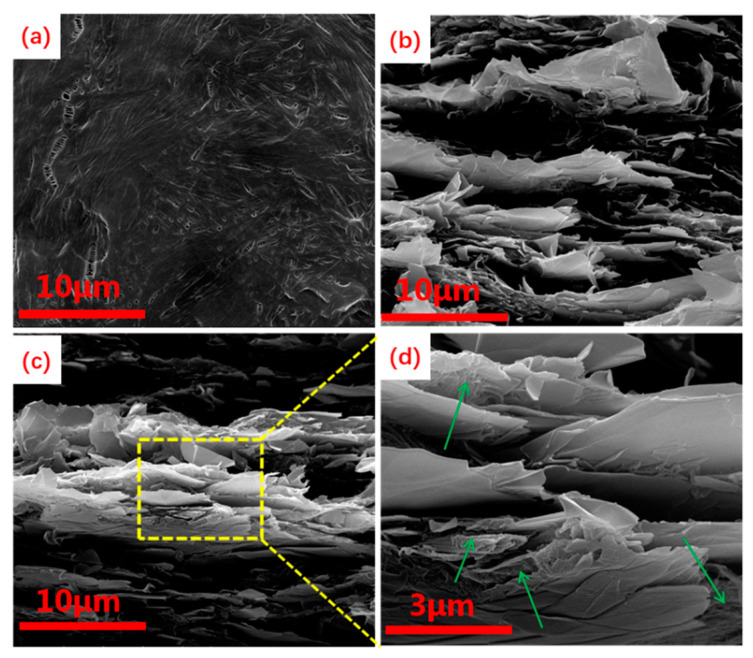
(**a**) SEM image of the cross section of PEO/CNF-FLG 0%; (**b**) SEM image of the cross section of PEO/CNF-FLG 50%; (**c**) SEM image of the cross section of PEO/CNF-FLG 80%; (**d**) SEM image of the cross section of PEO/CNF-FLG 80% at high magnification. The green arrows indicate CNFs.

The fracture morphologies of PEO/CNFs-FLG nanocomposite films with different CNFs-FLG content were visualized by SEM. The pure PEO film (Figure 3a) displayed a comparatively smooth fracture morphology with some microcracks due to the nature of PEO [42]. After blending with the CNFs-FLG filler, a layered structure could be observed (Figure 3b), indicating that the CNFs-FLG was uniformly distributed in the PEO matrix. However, with an increase in the proportion of CNFs-FLG nanofiller in the PEO, some obvious agglomeration was observed (Figure 3c,d). As indicated by the green arrows in Figure 3d, the CNFs coupled with the PEO could effectively wrap the FLG. Notably, it is possible for the hydroxyl groups on CNFs to form hydrogen bonding with the ether oxygen on PEO [43], which could improve the compatibility between PEO and CNFs-FLG.

### 3.3. Thermal Analysis

**Figure 4:**
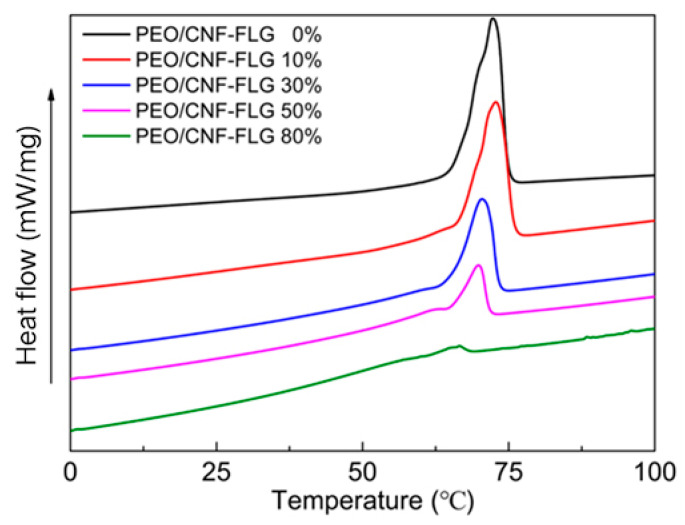
DSC curves of various PEO/CNFs-FLG composites.

The DSC curves of the PEO/CNFs-FLG composites with different CNFs-FLG filler content are shown in Figure 4. The melting point (Tm), melting enthalpy (ΔH_f_) and crystallinity (X) were calculated, and the results are provided in Table 2. The melting point of the pure PEO was 72.3 °C, and its crystallinity was 84.6%. With the increase in CNFs-FLG content, both the melting point and the crystallinity of PEO decreased. This is because the CNFs-FLG hybrid fillers were dispersed in the PEO matrix and a large proportion of the -OH on the CNFs and of the -OH and -COOH on the FLG could form strong interfacial bonding with the oxygen-containing groups of the PEO through hydrogen bonding. This hindered the ordered arrangement of PEO molecular chains in the crystallization process [43], resulting in the decrease in crystallinity.

### 3.4. Mechanical Properties

**Figure 5:**
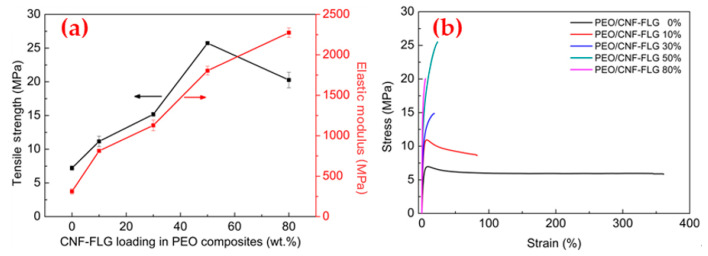
(**a**) Tensile strength and Young’s modulus of PEO/CNFs-FLG nanocomposite films; (**b**) Stress-strain curves of PEO/CNFs-FLG nanocomposite films.

Figure 5 compares the mechanical properties of the PEO/CNFs-FLG nanocomposite films with different CNFs-FLG content. As shown in Figure 5a, when the content of CNFs-FLG nanofillers was 50 wt.%, the tensile strength and Young’s modulus of the composite were 25.7 MPa and 1804.7 MPa, 2.6 and 4.8 times higher than those of pure PEO, respectively. Such remarkable reinforcement might be explained from two aspects. On one hand, both CNFs and FLG themselves have excellent mechanical properties and have been well utilized as reinforcing fillers [41]. On the other hand, the CNFs and FLG were bonded by hydrogen bonding and hydrophobic interaction, while a large number of hydroxyl groups exposed on the CNFs could interact with the -O- group on the PEO molecules through hydrogen bonding, giving rise to an effective stress transfer at the interface. Therefore, when the material was deformed (e.g., subjected to stretching), the external force could be effectively transferred to the strong CNFs and FLG via the hydrogen bonds, giving rise to enhanced mechanical properties. However, when the CNFs-FLG content was too high (PEO/CNF-FLG 80%), the CNFs-FLG fillers tended to agglomerate in the polymer matrix, which formed structural defects. In the meantime, the hydrogen bonding also became limited with lower PEO content. Therefore, the tensile strength of the film showed a slight decrease.

**Figure 6:**
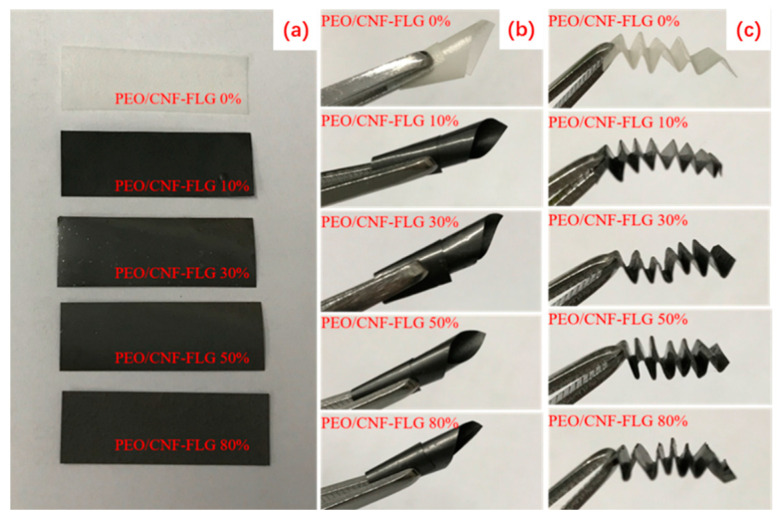
Digital photos of PEO/CNFs-FLG composite films under different deformations: flat (**a**); twist (**b**); fold (**c**).

It should be noted that the addition of CNFs-FLG reduced the elongation at break of the composite films (Figure 5b). Nonetheless, they all exhibited excellent flexibility and foldability. As demonstrated in Figure 6, all the composite films could be easily twisted or folded without any cracking.

### 3.5. Electrical Conductivity

**Figure 7:**
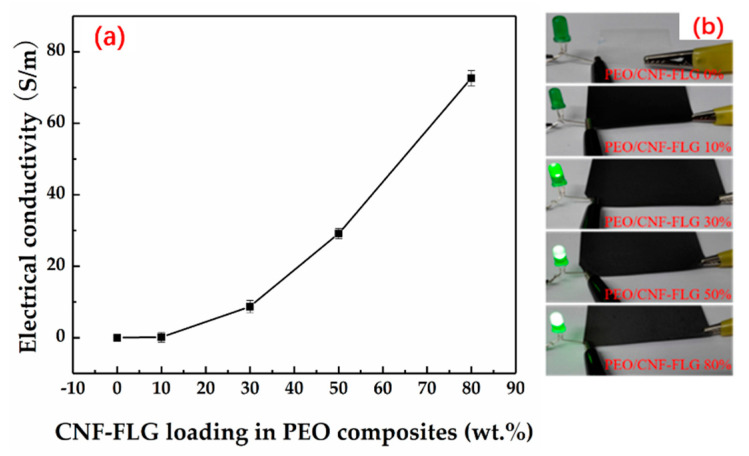
(**a**) Electrical conductivity of various PEO/CNFs-FLG composite films; (**b**) Lighting experiment with green LED.

The electrical conductivity of the various PEO/CNFs-FLG composite films was measured according to Equations (1) and (2), and the results are shown in Figure 7. The pure PEO film did not conduct electricity at all. When the CNFs-FLG content was 10 wt.%, the conductivity of the composite film was only 0.12 S/m, which is in the category of non-conductive materials. With the increase in CNFs-FLG content, the conductivity of the composite film was significantly improved. This is normal as the electrical conductivity is mainly governed by the content of conductive filler. A high loading of conductive filler helps to form a more complete conductive network inside the composite. These results agreed well with LED lamp lighting tests. As shown in Figure 7b, a green LED lamp was connected with the composite film and a power supply to form a circuit. When the CNFs-FLG content was 0 or 10 wt.%, the LED lamp did not light up. With the increase in CNFs-FLG content, the brightness of the LED lamp gradually increased, suggesting an improved conductive network with higher FLG additions. When the CNFs-FLG filler content was 80 wt.% (FLG only was 40 wt.%), the conductivity of the composite film reached its highest level of 72.6 S/m.

## 4. Conclusions

In this work, a facile and effective strategy was developed for the preparation of highly dispersed FLG in water and the production of strong and conductive PEO composite films with the obtained filler. Wet co-milling of bamboo pulp and EG was verified to be quite effective in simultaneously peeling off CNFs and FLG from their precursors. The resultant CNFs were crucial for the dispersion of the in situ-generated FLG in water owing to their hydrogen bonding and hydrophobic–hydrophobic interactions. When the obtained CNFs-FLG suspension was added to a PEO solution to make PEO/CNFs-FLG composite films, remarkably improved properties were realized. The tensile strength and Young’s modulus of the PEO/CNFs-FLG 50% composite film were 2.6 and 4.8 times those of pure PEO, respectively. Additionally, with the incorporation of FLG, the electrical conductivity of the PEO/CNFs-FLG films also sharply increased. Due to the excellent mechanical and electrical properties, we foresee that PEO/CNFs-FLG composite films may find broad applications in sensor, supercapacitor and electromagnetic shielding materials and so on.

## Figures and Tables

**Table 1:** Formulation of PEO/CNFs-FLG composites.

Samples	PEO (g)	Water (g)	CNFs-FLG (g)	CNFs-FLG Dispersion (g, 1wt.%)
PEO/CNFs-FLG 0%	1	50	0	0
PEO/CNFs-FLG 10%	0.9	45	0.1	10
PEO/CNFs-FLG 30%	0.7	35	0.3	30
PEO/CNFs-FLG 50%	0.5	25	0.5	50
PEO/CNFs-FLG 80%	0.2	10	0.8	80

**Table 2:** T_m_, ΔH_f_ and degree of crystallinity for various PEO/CNF-FLG composites.

Samples	T_m_ (°C)	ΔH_f_ (J/g)	X (%)
PEO/CNFs-FLG 0%	72.3	180.7	84.6
PEO/CNFs-FLG 10%	72.8	143.3	74.5
PEO/CNFs-FLG 30%	70.4	95.2	63.6
PEO/CNFs-FLG 50%	69.8	57.9	54.2
PEO/CNFs-FLG 80%	66.5	25.6	59.9

## Data Availability

The data presented in this study are available within this article. Further inquiries may be directed to the authors.
